# Evaluation of variation in the phosphoinositide-3-kinase catalytic subunit alpha oncogene and breast cancer risk

**DOI:** 10.1038/bjc.2011.448

**Published:** 2011-10-27

**Authors:** K N Stevens, M Garcia-Closas, Z Fredericksen, M Kosel, V S Pankratz, J L Hopper, G S Dite, C Apicella, M C Southey, M K Schmidt, A Broeks, L J Van ‘t Veer, R A E M Tollenaar, P A Fasching, M W Beckmann, A Hein, A B Ekici, N Johnson, J Peto, I dos Santos Silva, L Gibson, E Sawyer, I Tomlinson, M J Kerin, S Chanock, J Lissowska, D J Hunter, R N Hoover, G D Thomas, R L Milne, JI Arias Pérez, A González-Neira, J Benítez, B Burwinkel, A Meindl, R K Schmutzler, C R Bartrar, U Hamann, Y D Ko, T Brüning, J Chang-Claude, R Hein, S Wang-Gohrke, T Dörk, P Schürmann, M Bremer, P Hillemanns, N Bogdanova, J V Zalutsky, Y I Rogov, N Antonenkova, A Lindblom, S Margolin, A Mannermaa, V Kataja, V-M Kosma, J Hartikainen, G Chenevix-Trench, X Chen, P Peterlongo, B Bonanni, L Bernard, S Manoukian, X Wang, J Cerhan, C M Vachon, J Olson, G G Giles, L Baglietto, C A McLean, G Severi, E M John, A Miron, R Winqvist, K Pylkäs, A Jukkola-Vuorinen, M Grip, I Andrulis, J A Knight, G Glendon, A M Mulligan, A Cox, I W Brock, G Elliott, S S Cross, P P Pharoah, A M Dunning, K A Pooley, M K Humphreys, J Wang, D Kang, K-Y Yoo, D-Y Noh, S Sangrajrang, V Gabrieau, P Brennan, J McKay, H Anton-Culver, A Ziogas, F J Couch, D F Easton, the GENICA Network, kConFab Investigators, Australian Ovarian Cancer Study Group

**Affiliations:** 1 Department of Health Sciences Research, Mayo Clinic, 200 First Street SW, Rochester, MN 55905, USA; 2 Sections of Epidemiology and Genetics, Institute of Cancer Research and Breakthrough Breast Cancer Research Centre, 123 Old Brompton Road, London SW7 3RP, UK; 3 Centre for Molecular, Environmental, Genetic and Analytic Epidemiology, The University of Melbourne, Victoria 3010, Australia; 4 Department of Pathology, The University of Melbourne, Victoria 3010, Australia; 5 The Netherlands Cancer Institute, Plesmanlaan 121, 1066 CX, Amsterdam, The Netherlands; 6 Leiden University Medical Center, Albinusdreef 2, 2333 ZA, Leiden, The Netherlands; 7 Division of Hematology and Oncology, Department of Medicine, University of California at Los Angeles, David Geffen School of Medicine, Los Angeles, CA 90095, USA; 8 Department of Gynecology and Obstetrics, University Breast Center Franconia, Friedrich-Alexander University Erlangen-Nuremberg, Comprehensive Cancer Center Erlangen-Nuremberg, Schloßplatz 4, 91054 Erlangen, Germany; 9 Institute of Human Genetics, Friedrich-Alexander University Erlangen-Nuremberg, Schloßplatz 4, 91054 Erlangen, Germany; 10 Breakthrough Breast Cancer Research Centre, Institute of Cancer Research, 123 Old Brompton Road, London SW7 3RP, UK; 11 Faculty of Epidemiology and Population Health, London School of Hygiene and Tropical Medicine, Keppel Street, London WC1E 7HT, UK; 12 National Institute for Health Research (NIHR) Comprehensive Biomedical Research Centre, Guy's and St Thomas' NHS Foundation Trust in partnership with King's College London and King's College Hospital NHS Foundation Trust, Guy's Hospital, Great Maze Pond, London SE19RT, UK; 13 Oxford Biomedical Research Centre, Molecular and Population Genetics, Wellcome Trust Centre for Human Genetics, University of Oxford, Oxford OX3 7BN, UK; 14 NUIG Department of Surgery, Clinical Science Institute, University Hospital Galway, Galway 091 524 411, Ireland; 15 Division of Cancer Epidemiology and Genetics, National Cancer Institute, 6120 Executive Boulevard, MSC 7242, Bethesda, MD 20892-7335, USA; 16 Department of Cancer Epidemiology and Prevention, M. Sklodowska-Curie Memorial Cancer Center, Institute of Oncology, 5 Roentgena Street, 02-781 Warsaw, Poland; 17 Program in Molecular and Genetic Epidemiology, Harvard School of Public Health, 677 Huntington Avenue, Boston, MA 02115, USA; 18 Genetic and Molecular Epidemiology Group, Spanish National Cancer Research Centre (CNIO), Calle Melchor Fernández Almagro 3, Madrid 28029, Spain; 19 Servicio de Cirugía General y Especialidades, Hospital Monte Naranco, Av. Doctores Fernández Vega 107, Oviedo 33012, Spain; 20 Human Gentyping Unit (CeGen), Spanish National Cancer Research Centre (CNIO), Madrid, Spain; 21 Human Genetics Group, Spanish National Cancer Research Centre (CNIO), Calle Melchor Fernández Almagro 3, Madrid 28029, Spain; 22 Department of Obstetrics and Gynecology, University Hospital Heidelberg, Im Neuenheimer Feld 280, D-69120 Heidelberg, Germany; 23 Molecular Epidemiology Group, German Cancer Research Center (DKFZ), Im Neuenheimer Feld 280, D-69120 Heidelberg, Germany; 24 Department of Gynaecology and Obstetrics, Technical University of Munich, Arcisstraße 21, 80333 Munich, Germany; 25 Division of Molecular Gyneco-Oncology, Department of Gynaecology and Obstetrics, Center of Molecular Medicine Cologne (CMMC), University Hospital of Cologne, 50931 Cologne, Germany; 26 Institute of Human Genetics, University of Heidelberg, Im Neuenheimer Feld 366, 69120 Heidelberg, Germany; 27 Deutsches Krebsforschungszentrum (DKFZ), Im Neuenheimer Feld 280, D-69120 Heidelberg, Germany; 28 Department of Internal Medicine, Evangelische Kliniken Bonn gGmbH, Betriebsstätte Johanniter Krankenhaus, Johanniterstraße 3-5; 53113 Bonn, Germany; 29 Institute for Prevention and Occupational Medicine of the German Social Accident Insurance (IPA), Burkle-de-la-Camp-Platz 1, 44789 Bochum, Germany; 30 Division of Cancer Epidemiology, German Cancer Research Center (DKFZ), Im Neuenheimer Feld 280, D-69120 Heidelberg, Germany; 31 Department of Obstetrics and Gynecology, University of Ulm, Albert-Einstein-Allee 11, 89081 Ulm, Germany; 32 Hannover Medical School, Gynaecology Research Unit, 30625 Hannover, Germany; 33 Hannover Medical School, Clinics of Radiation Oncology, 30625 Hannover, Germany; 34 Hannover Medical School, Clinics of Obstetrics and Gynaecology, 30625 Hannover, Germany; 35 Department of Molecular Medicine and Surgery, Karolinska Institutet, S17176 Stockholm, Sweden; 36 Department of Oncology Pathology, Karolinska Institutet, S17176 Stockholm, Sweden; 37 Institute of Clinical Medicine, Department of Pathology, University of Eastern Finland and Kuopio University Hospital, Biocenter Kuopio, FI-70211 Kuopio, Finland; 38 Institute of Clinical Medicine, Department of Oncology, University of Eastern Finland, Kuopio University Hospital, FI-70211 Kuopio, Finland; 39 Queensland Institute of Medical Research, 300 Herston Road, Herston, QLD 4029, Australia; 40 Unit of Molecular Bases of Genetic Risk and Genetic Testing, Department of Preventive and Predicted Medicine, Fondazione IRCCS Istituto Nazionale Tumori (INT) and IFOM, Fondazione Istituto FIRC di Oncologia Molecolare; via Adamello, 16 - 20139 Milano, Italy; 41 Division of Cancer Prevention and Genetics, Istituto Europeo di Oncologia, via Ripamonti 435, 20141 Milano, Italy; 42 Department of Experimental Oncology, Istituto Europeo di Oncologia and Consortium for Genomics Technology (Cogentech); via Adamello, 16 - 20139 Milano, Italy; 43 Unit of Medical Genetics, Department of Preventive and Preventive Medicine, Fondazione IRCCS Istituto Nazionale Tumori (INT), via Venezian 1 - 20133 Milano, Italy; 44 Cancer Epidemiology Centre, Cancer Council Victoria, 1 Rathdowne Street, Carlton VIC 3053, Australia; 45 Centre for Molecular, Environmental, Genetic, and Analytic Epidemiology, The University of Melbourne, Level 4, 207 Bouverie Street, Victoria 3010, Australia; 46 Department of Anatomical Pathology, The Alfred Hospital, Commercial Road, Prahran VIC 3181, Australia; 47 Department of Epidemiology, Cancer Prevention Institute of California, 2201 Walnut Avenue, Suite 300, Fremont, CA 94538, USA; 48 Department of Surgery, Dana-Farber Cancer Institute, 450 Brookline Avenue, Boston, MA 02215-5450, USA; 49 Laboratory of Cancer Genetics, Department of Clinical Genetics and Biocenter Oulu, University of Oulu, Oulu University Hospital, Aapistie 5 A, 90220 Oulu, Finland; 50 Department of Oncology, University of Oulu, Oulu University Hospital, Aapistie 5 A, 90220 Oulu, Finland; 51 Department of Surgery, University of Oulu, Oulu University Hospital, Aapistie 5 A, 90220 Oulu, Finland; 52 Ontario Cancer Genetics Network, Cancer Care Ontario, 620 University Avenue, Toronto, ON M5G 2C1 ON, Canada; 53 Fred A. Litwin Center for Cancer Genetics, Mount Sinai Hospital, 600 University Avenue, Toronto, ON M5G 1X5, ON Canada; 54 Department of Molecular Genetics, University of Toronto, 105 George Street, Toronto, ON M5A 2N4 ON, Canada; 55 Samuel Lunenfeld Research Institute, Mount Sinai Hospital, 600 University Avenue, Toronto, ON M5G 1X5 ON, Canada; 56 Division of Epidemiology, Dalla Lana School of Public Health, University of Toronto, 105 George Street, Toronto, ON M5A 2N4 ON, Canada; 57 Department of Laboratory Medicine and Pathobiology, University of Toronto, 105 George Street, Toronto, ON M5A 2N4 ON, Canada; 58 Department of Laboratory Medicine and Keenan Research Centre of the Li Ka Shing Knowledge Institute, St Michael's Hospital, 30 Bond Street, Toronto, ON M5B 1W8 ON, Canada; 59 Faculty of Medicine, Dentistry and Health, University of Sheffield, Beech Hill Road, Sheffield S102RX, UK; 60 Medical Genetics Research Group, Faculty of Medicine and Human Sciences, University of Manchester, Oxford Road, Manchester M13 9PL, UK; 61 Department of Neuroscience, Faculty of Medicine, Dentistry and Health, University of Sheffield, Beech Hill Road, Sheffield S102RX, UK; 62 United Kingdom Department of Oncology and Department of Public Health and Primary Care University of Cambridge, Cambridge, UK; 63 Department of Oncology, University of Cambridge, Robinson Way, Cambridge CB2 0RE, UK; 64 Centre for Cancer Genetic Epidemiology, University of Cambridge, Worts Causeway, Cambridge CB1 8RN, UK; 65 Seoul National University College of Medicine, Yongon _103 Daehak-ro, Jongno-gu, Seoul 110-799, Korea; 66 National Cancer Institute, Rama 6 Road, 10400 Bangkok, Thailand; 67 International Agency for Research on Cancer, 150 Cours Albert Thomas, 69372 Lyon CEDEX 08, France; 68 Department of Epidemiology, School of Medicine, University of California Irvine, 224 Irvine Hall, Irvine, CA 92697, USA; 69 Department of Laboratory Medicine and Pathology, Mayo Clinic, 200 First Street SW, Rochester, MN 55905, USA

**Keywords:** genetic susceptibility, neoplasms, association study

## Abstract

**Background:**

Somatic mutations in phosphoinositide-3-kinase catalytic subunit alpha (*PIK3CA*) are frequent in breast tumours and have been associated with oestrogen receptor (ER) expression, human epidermal growth factor receptor-2 overexpression, lymph node metastasis and poor survival. The goal of this study was to evaluate the association between inherited variation in this oncogene and risk of breast cancer.

**Methods:**

A single-nucleotide polymorphism from the *PIK3CA* locus that was associated with breast cancer in a study of Caucasian breast cancer cases and controls from the Mayo Clinic (MCBCS) was genotyped in 5436 cases and 5280 controls from the Cancer Genetic Markers of Susceptibility (CGEMS) study and in 30 949 cases and 29 788 controls from the Breast Cancer Association Consortium (BCAC).

**Results:**

Rs1607237 was significantly associated with a decreased risk of breast cancer in MCBCS, CGEMS and all studies of white Europeans combined (odds ratio (OR)=0.97, 95% confidence interval (CI) 0.95–0.99, *P*=4.6 × 10^−3^), but did not reach significance in the BCAC replication study alone (OR=0.98, 95% CI 0.96–1.01, *P*=0.139).

**Conclusion:**

Common germline variation in *PIK3CA* does not have a strong influence on the risk of breast cancer

Phosphatidylinositol-3 kinases (PI3Ks) constitute a lipid kinase family integral to signalling pathways that regulate many cancer-related processes, including cell proliferation, adhesion, apoptosis, survival and motility (Fruman *et al*, 1998; Cantley, 2002). Alteration of PI3K family members, such as amplification of the phosphoinositide-3-kinase catalytic subunit alpha (*PIK3CA*) oncogene on chromosome 3q26 that encodes the p110*α* catalytic subunit of PI3K, are commonly observed in human cancers. Amplification and overexpression of *PIK3CA* results in increased production of the phosphatidylinositol-3,4,5-triphosphate second messenger, hyperactivation of the PI3K/AKT pathway, and stimulation of cellular transformation and tumour progression (Shayesteh *et al*, 1999; Ma *et al*, 2000; Fresno Vara *et al*, 2004; Saal *et al*, 2005; Samuels and Ericson, 2006). Somatic mutations in *PIK3CA* are also common in colon (18–32%), gastric (4–25%), endometrial (36%), liver (36%), brain (27%) and breast (18–40%) tumours (Bachman *et al*, 2004; Campbell *et al*, 2004; Samuels *et al*, 2004; Karakas *et al*, 2006; Ligresti *et al*, 2009). Functional analyses have shown that many of these mutations activate PIK3CA enzymatic activity and stimulate downstream AKT signalling, promoting growth factor-independent growth and metastasis (Samuels *et al*, 2004; Samuels and Ericson, 2006).

In breast tumours, *PIK3CA* mutations have been consistently associated with ER-positive and human epidermal growth factor receptor-2 (HER2)-positive tumour status (Saal *et al*, 2005; Li *et al*, 2006; Perez-Tenorio *et al*, 2007; Stemke-Hale *et al*, 2008) (Saal *et al*, 2005; Perez-Tenorio *et al*, 2007). The correlation between these mutations and breast cancer prognosis is less clear, with several studies reporting associations between *PIK3CA* mutations and lymph node metastasis and worse overall and breast cancer-specific survival (Saal *et al*, 2005; Li *et al*, 2006; Lai *et al*, 2008; Aleskandarany *et al*, 2010), whereas other studies have reported associations with longer survival particularly among patients with ER-positive, HER2-negative tumours (Perez-Tenorio *et al*, 2007; Kalinsky *et al*, 2009; Loi *et al*, 2010).

Although the pathological and clinical significance of *PIK3CA* somatic mutations has been well studied, the contribution of inherited variation in this important oncogene to risk of breast cancer is unknown. Here we investigated the influence of germline variation in *PIK3CA* on breast cancer risk.

## Materials and Methods

### Mayo clinic breast cancer study

The details of the Mayo Clinic Breast Cancer case–control Study (MCBCS) have been described previously (Wang *et al*, 2008). Briefly, cases were comprised of Caucasian women with invasive breast cancer diagnosed within 6 months of ascertainment with no prior history of cancer. Controls were comprised of Caucasian women visiting the Mayo Clinic for general medical exams in the Department of Internal Medicine with no prior history of cancer. Participants were recruited under an Institutional Review Board approved protocol. A total of 798 cases and 843 controls were utilised for stage 1 genotyping (Table 1).

### Replication studies

The Cancer Genetic Markers of Susceptibility (CGEMS) breast cancer case–control study and 26 case–control studies from Breast Cancer Association Consortium (BCAC) contributed data to these analyses (described in Supplementary Table 1). Stage 1 of the CGEMS GWAS included 1145 cases and 1142 controls of self-reported white European ancestry (Thomas *et al*, 2009), whereas the combined Stage 1 and 2 of CGEMS included a total of 5436 cases and 5280 controls (Table 1). The BCAC replication was comprised of 24 studies of women of primarily European descent (Supplementary Table 1), 1702 additional samples from MCBCS and two studies (SEBCS and TBCS) of women from Southeast Asia (Table 1). Final combined analyses included 35 991 breast cancer cases and 35 153 controls of white European ancestry, as well as 2183 breast cancer cases and 1469 controls of Asian ancestry. Study participants were recruited under protocols approved by the institutional review board at each institution and all subjects provided written informed consent.

### Genotyping

Four haplotype-tagging single-nucleotide polymorphisms (SNPs) within *PIK3CA* (rs13320527, rs3729692, rs1607237, rs9838117) were selected (*r*^2^>0.80 in European–American genotype data from HapMap release 21). A total of 1741 Mayo Clinic samples (798 cases, 843 controls and 100 duplicates) were genotyped on custom oligo pool assays at Illumina Corporation (San Diego, CA, USA) using the Illumina GoldenGate assay. All SNPs had genotype call rates >95%. Concordance between duplicate samples was 100%. Genotyping of rs1607237 in CGEMS and BCAC was performed using a TaqMan allelic discrimination assay or the Sequenom platform (Sequenom, San Diego, CA, USA) via standard protocols. Genotyping concordance was verified with internal duplicates and overall data quality was ensured using independent genotyping of 96 CEU samples by each genotyping center (Garcia-Closas *et al*, 2008). All studies met the specified criteria for call rate (>95%).

### Pathology and tumour markers

The collection of pathology and tumour marker information for BCAC has been described previously (Yang *et al*, 2011). Pathology data were also available for 900 CGEMS subjects. Briefly, studies provided information on histopathological subtype, grade of differentiation, tumour size, nodal involvement and stage at diagnosis of breast tumours. All studies except BBCS, GC-HBOC and HMBCS provided data on ER and progesterone receptor (PR) status of tumours, and 12 studies provided data on HER2 (Supplementary Table 2). ER/PR status was most commonly defined using data from medical records. Oestrogen receptor and PR negative status was defined as <10% of the tumour cells stained. Human epidermal growth factor receptor-2-negative status was typically defined as a score of 0 or 1+ on a HER2 immunohistochemistry (IHC) scale of 0–3+.

### Statistical methods

Evidence of departure from Hardy–Weinberg equilibrium (HWE) was assessed in controls using a goodness of fit test and none was observed (HWE *P*⩾0.001). Single-nucleotide polymorphism associations were tested using unconditional logistic regression adjusting for age and state of residence in a log-additive model. We also calculated odds ratios (ORs) and 95% confidence intervals (CIs) separately for heterozygotes and rare homozygotes. The association between rs1607237 and breast cancer risk in stage 1 of the CGEMS GWAS was evaluated as previously described (Thomas *et al*, 2009). Associations with breast cancer risk in the BCAC studies and the combined BCAC, MCBCS and CGEMS studies were evaluated using unconditional logistic regression adjusting for study center. A likelihood ratio test of heterogeneity by age groups was not significant (*P*=0.10), and further adjustment for age did not change the results. Analyses of pathology-specific subsets of cases were conducted using polytomous regression with controls as the reference outcome, adjusting for study site.

## Results

Of four *PIK3CA* haplotype-tagging SNPs, rs1607237 was significantly associated with risk of breast cancer in MCBCS (OR=0.85, 95% CI 0.73–0.98, *P*=0.023; Table 2, Supplementary Figure 1). Next we evaluated associations between rs1607237 and breast cancer risk in 1145 cases and 1142 controls genotyped in stage 1 of the CGEMS breast cancer GWAS (Thomas *et al*, 2009). Rs1607237 was significantly associated with breast cancer risk (heterozygous OR=1.12, homozygous OR=0.79, score *P*=0.017). To provide a more stable estimate of risk in this population, 8429 additional CGEMS subjects were genotyped for rs1607237. In all 5436 cases and 5280 controls from stage 1 and 2 of CGEMS, rs1607237 was strongly associated with a decrease in breast cancer risk (OR=0.92, 95% CI 0.88–0.98, *P*=0.0050; Table 2).

This finding provided the rationale for further evaluation of this SNP in 23 BCAC studies involving women of European ancestry (28 766 cases, 28 319 controls), and two BCAC studies of Asian women (2183 cases, 1469 controls; Table 1). Rs1607237 was not significantly associated with breast cancer risk in the 23 BCAC studies of women of European ancestry (OR=0.98, 95% CI 0.96–1.01, *P*=0.139) or in the two Asian BCAC studies (OR=1.05, 95% CI 0.94–1.16, *P*=0.39; Table 2). However, when combining all genotype data from the three stages of this study (MCBCS, CGEMS and BCAC; Supplementary Table 3), rs1607237 was significantly associated with risk of breast cancer (OR=0.97, 95% CI 0.95–0.99, *P*=9.5 × 10^−3^). Similarly, a significant association was observed when considering only women of European ancestry in the combined analysis (OR=0.97, 95% CI 0.95–0.99, *P*=4.6 × 10^−3^; (Table 2). There was no evidence of heterogeneity by study site among the 25 Caucasian studies (*P*=0.14; Supplementary Figure 2).

To further understand the association with breast cancer, we restricted the analysis to women with invasive breast cancer. Rs1607237 was associated with a reduced risk of invasive breast cancer (OR=0.97, 95% CI 0.95–0.99, *P*=0.012; Table 2), whereas no association with risk of ductal carcinoma *in situ* was observed (OR=0.93, 95% CI 0.85–1.02, *P*=0.12). In addition, we explored differences in *PIK3CA* SNP associations in the combined data set by tumour subtype (Supplementary Table 4). The rs1607237 variant was not associated with any subtypes defined by ER, PR or HER2 status, although it is important to note the reduction in sample size when restricting to these tumour subtypes.

## Discussion

Here we report an association between inherited variation in the oncogene *PIK3CA* and risk of breast cancer in a large, three-stage analysis utilising nearly 75 000 subjects from 27 case–control study studies. We show that rs1607237 is significantly associated with a small decrease in breast cancer risk (OR=0.97, 95% CI 0.95–0.99, *P*=9.5 × 10^−3^) in all studies combined and when considering only women of European ancestry in the combined studies (OR=0.97, 95% CI 0.95–0.99, *P*=4.6 × 10^−3^). However, the association did not achieve significance in the large third stage involving only BCAC studies. Although the first two stages of our analysis suggest an association between *PIK3CA* and breast cancer risk, our inability to confirm this finding in the BCAC studies suggests that the result should be interpreted with caution.

We further explored the linkage disequilibrium patterns in the *PIK3CA* coding and promoter regions to better understand the relationship between rs1607237 and other variation in this region. Rs1607237 was not in strong linkage disequilibrium with two non-synonymous polymorphic variants in the coding region of *PIK3CA*, rs1051399 (*r*^2^=0.0060) and rs3729680 (*r*^2^=0.034), which had been genotyped in HapMap samples of European ancestry. However, an additional 18 non-synonymous variants were either not polymorphic or had not been genotyped in the HapMap samples, making inference about the relationship between rs1607237 and all variants of unknown significance in the *PIK3CA* coding region difficult. In addition, two *PIK3CA* promoter SNPs were in low LD with rs1607237 (rs9831234, *r*^2^=0.16; rs2865084, *r*^2^=0.038). However, it remains possible that *PIK3CA* promoter SNPs that were not captured in this study are related to breast cancer risk.

It is also important to note that the effect estimate for rs1607237 in the BCAC replication studies and in the overall BCAC, MCBCS and CGEMS studies is quite small (OR=0.97). This limits our statistical power to detect significant associations in these studies despite the large sample size, particularly in analyses utilising pathology information that is available for only a subset of subjects. Similarly, we had limited power to detect associations in the original MCBCS study with the three non-significant *PIK3CA* SNPs. Thus, it remains possible that evaluation of these variants in the larger BCAC cohort might detect associations with risk. While the effect of rs1607237 on risk is small, the association between inherited variation in this important oncogene and breast cancer risk does provide valuable biological insight into the development of this disease. Validation of rs1607237 in GWAS studies from other large collaborative groups and additional studies by BCAC with detailed pathology information are necessary to confirm this association. Functional evaluation of this variant is needed to fully understand the relationship between inherited *PIK3CA* variation and breast cancer risk.

## Figures and Tables

**Table 1:** Studies contributing to evaluation of associations between rs1607237 and breast cancer risk

**Study** a	**Country**	**Cases *n* (%)**	**Controls *n* (%)**
ABCFS	Australia	1199 (3.1)	438 (1.2)
ABCS	The Netherlands	1465 (3.8)	548 (1.5)
BBCC	Germany	1060 (2.8)	994 (2.7)
BBCS	UK	1153 (3.0)	831 (2.3)
BIGGS	Ireland	1060 (2.8)	900 (2.5)
CGEMS^b^	USA	5436 (14.2)	5280 (14.4)
CNIO-BCS	Spain	752 (2.0)	823 (2.2)
GC-HBOC	Germany	864 (2.3)	1224 (3.3)
GENICA	Germany	1013 (2.7)	1012 (2.8)
GESBC	Germany	563 (1.5)	564 (1.5)
HABCS	Germany	1046 (2.7)	998 (2.7)
HMBCS	Belarus	1760 (4.6)	1015 (2.8)
KARBAC	Sweden	812 (2.1)	863 (2.4)
kConFab/AOCS	Australia/New Zealand	566 (1.5)	899 (2.5)
KBCP	Finland	485 (1.3)	427 (1.2)
MARIE	Germany	2754 (7.2)	5302 (14.5)
MBCSG	Italy	739 (1.9)	1231 (3.4)
MCBCS^c^	USA	1789 (4.7)	1554 (4.2)
MCCS	Australia	679 (1.8)	751 (2.1)
NC-BCFR	USA	388 (1.0)	154 (0.4)
OBCS	Finland	544 (1.4)	509 (1.4)
OFBCR	Canada	1170 (3.1)	329 (0.9)
SBCS	UK	1217 (3.2)	1201 (3.3)
SEARCH	UK	6520 (17.1)	6779 (18.5)
SEBCS^d^	Korea	1732 (4.5)	1178 (3.2)
TBCS^d^	Thailand	451 (1.2)	291 (0.8)
UCIBCS	USA	957 (2.5)	527 (1.4)
Total		38 174 (100)	36 622 (100)

a See Supplementary Table 1 for definition of study acronyms.

b Stage 2: Cancer Genetic Markers of Susceptibility study.

c Includes Stage 1: Mayo Clinic Breast Cancer Study.

d Asian case–control studies.

**Table 2:** Associations between rs1607237 and breast cancer in MCBCS, CGEMS and BCAC

					**2-d.f. model**	
			**Log-additive model**		**Heterozygous**	**Homozygous**
	**Cases**	**Controls**	**OR (95% CI)**	***P*-value**	**OR (95% CI)**	**OR (95% CI)**
Stage 1: MCBCS	798	843	0.85 (0.73–0.98)	0.023	0.75 (0.60–0.93)	0.76 (0.57–1.01)
Stage 2: CGEMS	5436	5280	0.92 (0.88–0.98)	0.0050	1.00 (0.92–1.09)	0.82 (0.73–0.92)
Stage 3: BCAC	28 766	28 319	0.98 (0.96–1.01)	0.139	0.96 (0.93–1.00)	0.97 (0.92–1.02)
Combined analysis	35 991	35 153	0.97 (0.95–0.99)	0.0046	0.97 (0.93–1.00)	0.94 (0.90–0.98)
Invasive	33 660	34 988	0.97 (0.95–0.99)	0.012	0.97 (0.94–1.00)	0.95 (0.90–0.99)
DCIS	1159	16 889	0.93 (0.85–1.02)	0.12	0.98 (0.85–1.12)	0.84 (0.70–1.02)

Abbreviations: BCAC=Breast Cancer Association Consortium; CGEMS=Cancer Genetic Markers of Susceptibility; CI=confidence interval; DCIS=ductal carcinoma *in situ*; MCBCS=Mayo Clinic breast cancer case–control study; OR=odds ratio.

## APPENDIX

### The GENICA Network

Gene Environment Interaction and Breast Cancer in Germany (GENICA): Dr. Margarete Fischer-Bosch-Institute of Clinical Pharmacology, Stuttgart, and University Tübingen, Germany (Hiltrud Brauch, Christina Justenhoven); Molecular Genetics of Breast Cancer, Deutsches Krebsforschungszentrum (DKFZ), Heidelberg, Germany (UH); Department of Internal Medicine, Evangelische Kliniken Bonn gGmbH, Johanniter Krankenhaus, Bonn, Germany (YDK, Christian Baisch); Institute of Pathology, Medical Faculty of the University of Bonn, Germany (Hans-Peter Fischer); Institute for Prevention and Occupational Medicine of the German Social Accident Insurance (IPA), Bochum, Germany (TB, Beate Pesch, Volker Harth, Sylvia Rabstein). The GENICA was funded by the Federal Ministry of Education and Research (BMBF) Germany grants 01KW9975/5, 01KW9976/8, 01KW9977/0 and 01KW0114, the Robert Bosch Foundation, Stuttgart, Deutsches Krebsforschungszentrum (DKFZ), Heidelberg, Institute for Prevention and Occupational Medicine of the German Social Accident Insurance (IPA), Bochum, as well as the Department of Internal Medicine, Evangelische Kliniken Bonn gGmbH, Johanniter Krankenhaus, Bonn, Germany.

*kConFab Investigators, Australian Ovarian Cancer Study Group*

Peter MacCallum Cancer Centre, St Andrews Place, East Melbourne VIC 3002, Australia.
