# Supplementary figures and images for: Evaluation of variation in the phosphoinositide-3-kinase catalytic subunit alpha oncogene and breast cancer risk
Source: Br J Cancer. 2011 Oct 27;105(12):1934–9. doi: 10.1038/bjc.2011.448 (PMC3251877; doi:10.1038/bjc.2011.448)

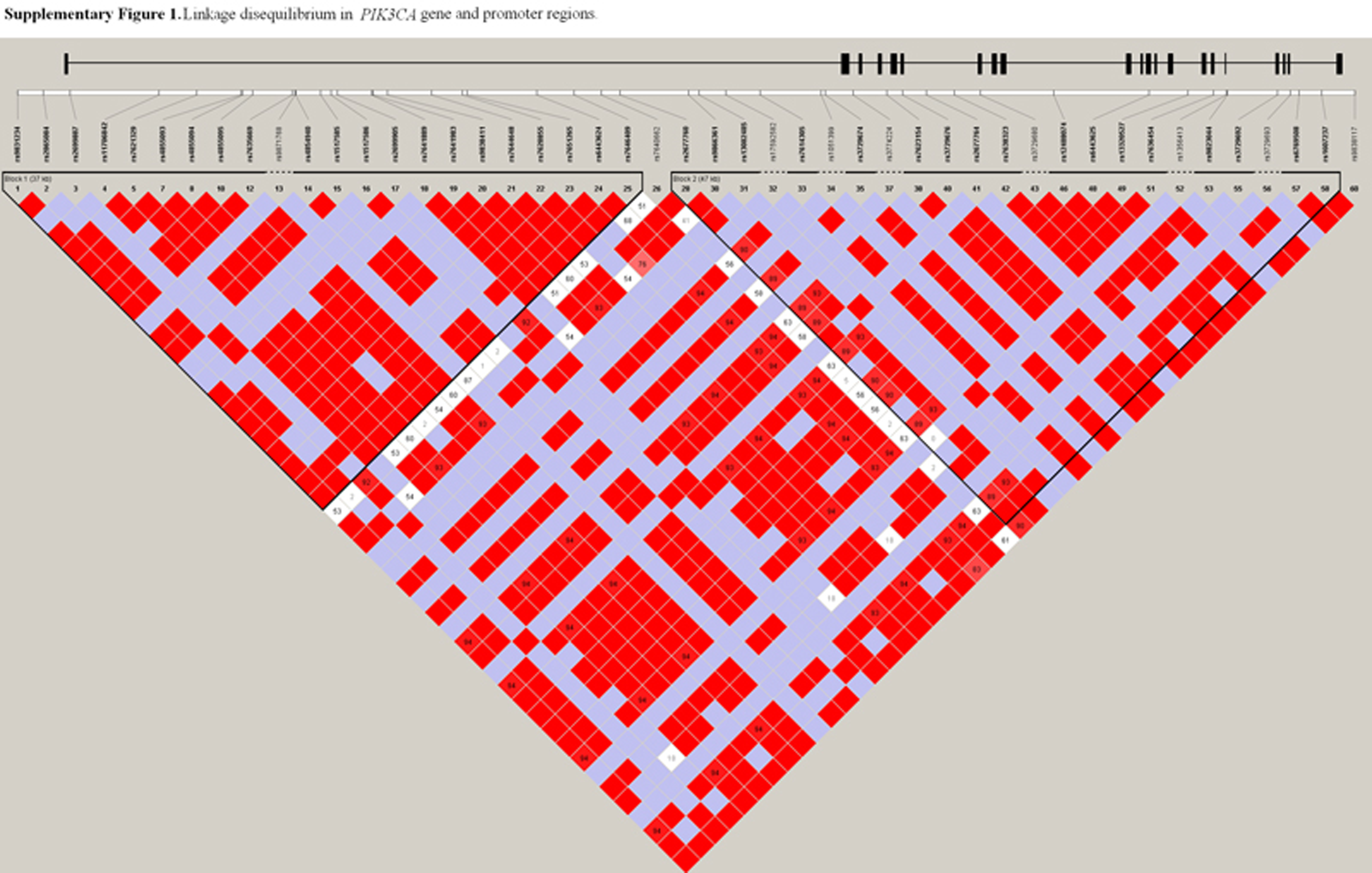

Supplement: Supplementary Figure 1 [file bjc2011448x1.tif]

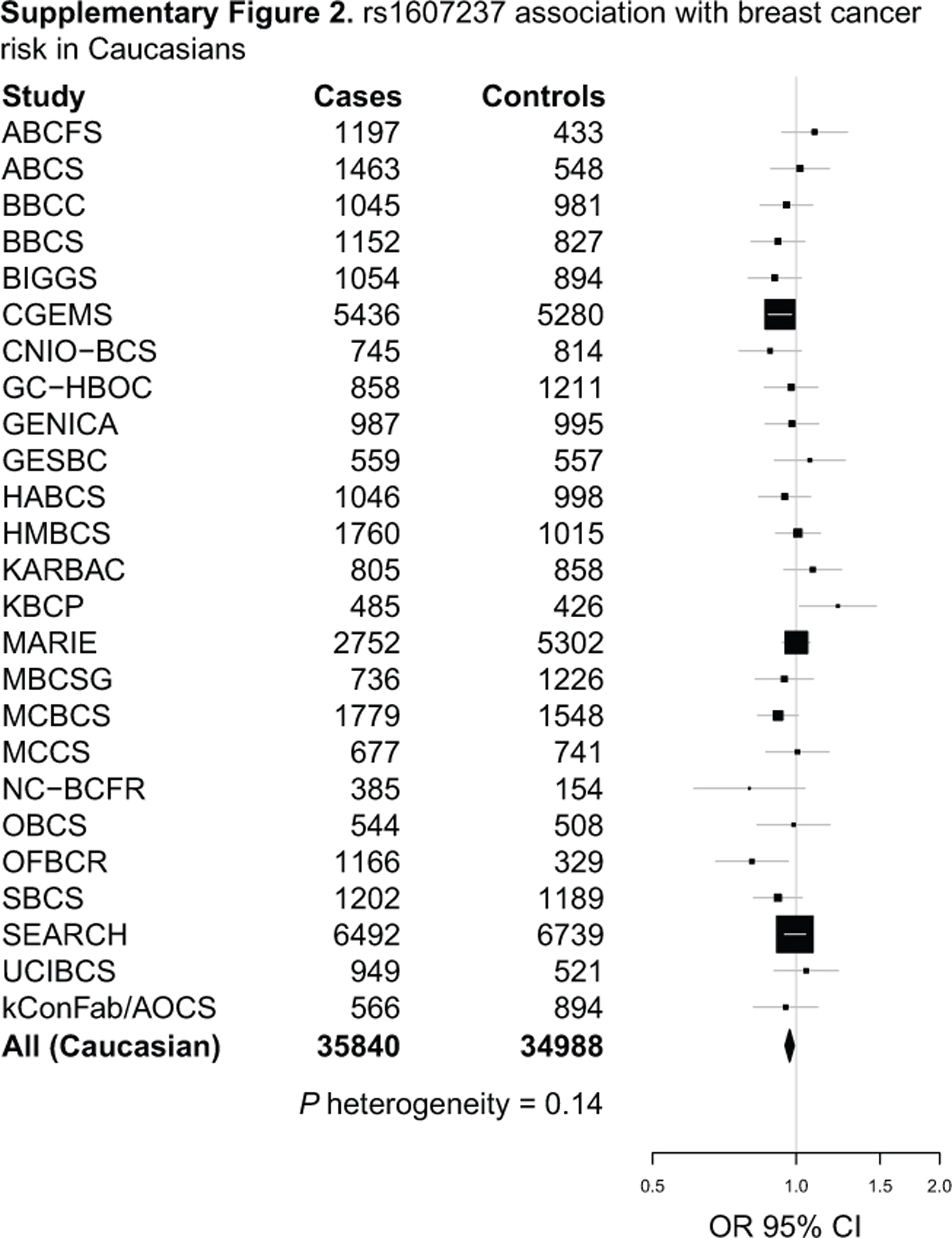

Supplement: Supplementary Figure 2 [file bjc2011448x2.tif]
