# Supplementary material for: Evaluation of variation in the phosphoinositide-3-kinase catalytic subunit alpha oncogene and breast cancer risk
Source: Br J Cancer. 2011 Oct 27;105(12):1934–9. doi: 10.1038/bjc.2011.448 (PMC3251877; doi:10.1038/bjc.2011.448)
Supplement: Supplementary Figure Legends [file bjc2011448x3.doc]

**Supplementary Figure legends**

**Supplementary Figure 1. Linkage disequilibrium in *PIK3CA***

A linkage disequilibrium (LD) plot is shown using 61 single nucleotide polymorphisms (SNPs) genotyped in HapMap subjects of European ancestry in the *PIK3CA* gene and promoter regions. High LD, measured by D’ equal to 1, is denoted by bright red squares. Intermediate LD is indicated by D’ values within white, pink, and red squares. Rs1607237 is located at the 3’ end of the gene (SNP 58) and is in high LD with the majority of the variation in *PIK3CA* and the *PIK3CA* promoter region. The PIK3CA gene is indicated above the LD plot, with exons and introns represented by black boxes and lines, respectively.

**Supplementary Figure 2. rs1607237 association with breast cancer risk in Caucasians**

Estimates for rs1607237 and risk of breast cancer among Caucasian women are shown by study. Study-specific odds ratios (95% CIs) are denoted by black boxes (black lines). Overall OR estimates are represented by black diamonds, where diamond width corresponds to 95% CI bounds. Box and diamond heights are inversely proportional to precision of the OR estimate.
