# Supplementary material for: Evaluation of variation in the phosphoinositide-3-kinase catalytic subunit alpha oncogene and breast cancer risk
Source: Br J Cancer. 2011 Oct 27;105(12):1934–9. doi: 10.1038/bjc.2011.448 (PMC3251877; doi:10.1038/bjc.2011.448)
Supplement: Supplementary Tables [file bjc2011448x4.doc]

**Supplementary Material**

**Supplementary Table 1.** **Descriptions of studies represented in replication stage**

| **Study** | **Abbreviation** | **Country** | **Study design** | **Definition of case patients** | **Definition of control subjects** | **Reported participation rates** | **Age at diagnosis, range (y)** |
| --- | --- | --- | --- | --- | --- | --- | --- |
| Australian Breast Cancer Family Study | ABCFS | Australia | Population-based | All cases diagnosed < age 40 plus a random sample of those diagnosed ages 40-59 from cancer registries in Victoria and New South Wales, plus a limited number diagnosed aged 60-69; cases living in Melbourne recruited from 1992-99 and in Sydney from 1993-98. | Identified from the electoral rolls in Melbourne from 1992-98 and Sydney from 1993-99. Frequency matched to cases by age in 5 year categories. | 75% of cases and 68% of controls completed questionnaires, 71% of cases and 55% of controls provided a blood sample | 23 - 69 |
| Amsterdam Breast Cancer Study | ABCS | Netherlands | Mixed | All cases (operable, invasive mammacarcinoma) aged <50 and diagnosed from 1974-1994 in 4 Dutch hospitals. | Random women <50 years of age at baseline from 2 population-based prospective studies - the Monitoring Project on Cardiovascular Risk Factors (1987-1991) and the Monitoring Project on Chronic Disease Risk Factors (1993-1997). These studies were run by National Institute for Public Health and the Environment, The Netherlands. Controls are selected from the same catchment area as cases. | 85% of cases and ~50% of controls for DNA (blood/paraffin). | 23 - 50 |
| Bavarian Breast Cancer Cases and Controls | BBCC | Germany | Mixed | Consecutive, unselected cases with invasive breast cancer recruited at the University Breast Centre, Franconia in Northern Bavaria during 2002-2006. | Healthy women with no diagnosis of cancer aged 55 or older. Invited by a newspaper advertisement in Northern Bavaria, and recruited during 2002-2006 | 95% of cases and 99% of controls provided a blood sample and an epidemiological questionnaire. | 22 - 96 |
| British Breast Cancer Study | BBCS | UK | 1) Cancer registry and 2) National Cancer Research network (NCRN) based cases; population based controls | 1) English & Scottish Cancer Registries: all breast cancer cases who developed a first primary before age 66 in 1971 or later and who subsequently developed a second primary cancer. 2) Breast cancer clinics: all breast cancer cases who developed a first primary before age 77 in 1967 or later and who either subsequently developed a second primary or had at least two affected female first degree relatives. | 68% of cases & 76% of controls provided a blood sample. | 68% of cases & 76% of controls provided a blood sample. | 24 - 76 |
| Breast Cancer in Galway Genetic Study | BIGGS | Ireland | Hospital-based | Unselected cases recruited from West of Ireland since 2001. Cases were recruited from University College Hospital Galway and surrounding hospitals | Women > 60 years with no personal history of any cancer and no family History of breast or ovarian cancer were identified from retirement groups in the West of Ireland (same catchment area as cases) during the period 2001-2008. | Not recorded | 24 - 90 |
| Cancer Genetic Markers of Susceptibility | CGEMS | USA | Prospective cohort study: nested case-control | Incident cases arising in the sub-cohort of 32,826 cohort members who gave a blood specimen in 1989-1990 are included if they were diagnosed with breast cancer prior to July 1, 2000. | Controls were women in this sub-cohort who were not diagnosed with breast cancer. Controls were matched to cases on age, postmenopausal status and postmenopausal hormone use. | All incident cases and selected controls are included. All cases and controls had provided a blood sample. | 44-79 |
| Spanish National Cancer Centre Breast Cancer Study | CNIO-BCS | Spain | Mixed | Two groups of cases:1) 574 consecutive breast cancer patients, unselected for family history, from 3 public hospitals, 2 in Madrid and one in Oviedo, from 2000 to 2005. 2) 291 cases with at least one first degree relative also affected with breast cancer, recruited through the CNIO family cancer clinic in Madrid from 2000 to 2004. | Women attending the Menopause Research Centre between 2000 and 2004 and female members of the College of Lawyers attending a free, targeted medical check-up in 2005, all free of breast cancer and all in Madrid | Not recorded. | 23 - 86 |
| German Consortium for Hereditary Breast & Ovarian Cancer | GC-HBOC | Germany | Mixed | Index patients from German breast cancer families; BRCA1/2 mutation free, collected 1996-2007 via Institute of Human Genetics, University Heidelberg & Department of Gynaecology & Obstetrics, Cologne & Department of Gynaecology and Obstetrics at the Ludwig-Maximilians-University, Munich; Germany. | Healthy, unrelated, ethnically matched female blood donors recruited in 2004 & 2007 by German Red Cross Blood Service of Baden-Württemberg-Hessen, Institute of Transfusion Medicine & Immunology, Mannheim. | Cases: About 3% refused to spend their blood samples for research purposes. Controls: Offers of blood donation from approximately 5% of donors were refused due to various reasons | 19 - 87 |
| Gene Environment Interaction and Breast Cancer in Germany | GENICA | Germany | Population-based | Incident breast cancer cases enrolled between 2000 and 2004 from the Greater Bonn area (by of the hospitals within the study region); all enrolled within 6 months of diagnosis | Selected from population registries from 31 communities in the greater Bonn area; matched to cases in 5-year age classes between 2001 and 2004 | Response rate 88% for cases and 67% for controls. Of these, DNA samples are available for 89% and 90% respectively. | 23 - 80 |
| Genetic Epidemiology Study of Breast Cancer by Age 50 | GESBC | Germany | Population-based | All incident cases diagnosed <50 years of age in 1992-5 in two regions: Rhein-Neckar-Odenwald and Freiburg, by surveying the 38 clinics serving these regions | Selected from random lists of residents of the study regions supplied by population registries; two controls were selected for each case, matched by age and study region. Recruitment was carried out 1992-1998. | 70.2% of cases and 61.2% of controls completed the questionnaire. | 24 - 50 |
| Hannover Breast Cancer Study | HABCS | Germany | Mixed | Cases who received radiotherapy for breast cancer at Hannover Medical School between 1997-2003, unselected for age or family history | Anonymous female blood bank donors at Hannover Medical School, collected from 8/2005-12/2005, with known age and ethnic background | Approx. 80% of cases and 70% of controls contacted agreed to give a blood sample | 25 - 91 |
| Hannover-Minsk Breast Cancer Study | HMBCS | Belarus | Mixed | Ascertainment at the Byelorussian Institute for Oncology and Medical Radiology Aleksandrov N.N. in Minsk or at one of 5 regional oncology centers in Gomel, Mogilev, Grodno, Brest or Vitebsk through the years 2002-2008. | Controls from the same population aged 18-72 years. Healthy (without personally history of cancer) female probunds recruited from the same geographical regions as cases during the years 2002-2008. About 75% of controls were women invited for general medical examination at five regional gynecology clinics (in Gomel, Mogilev, Grodno, Brest or Vitebsk ) and cancer-free volunteers ascertained at the Institute for Inherited Diseases in Minsk; 20% were cancer-free female blood bank donors recruited at Republic Blood Bank, Minsk, Belarus; finally 5% of controls were healthy cancer-free relatives of some breast cancer patients. | More than 60% for cases and more than 80% for controls. DNA available from all of the participants. | 16 - 82 |
| Karolinska Breast Cancer Study | KARBAC | Sweden | Mixed | 1. Familial cases from Department of Clinical Genetics, Karolinska University Hospital , Stockholm. 2. Consecutive cases from Department of Oncology, Huddinge & Söder Hospital, Stockholm 1998-2000 | Blood donors of mixed gender from same geographical region. Excess material was received from all blood donors over a 3 month period in 2004 (approximately 3000) and DNA was extracted from a random sample of 1500 | 1. NA 2. 70% of consecutive cases provided a blood-sample | 24 - 88 |
| Kuopio Breast Cancer Project | KBCP | Finland | hospital-based | Women seen at Kuopio University Hospital between 1990 and 1995 because of breast lump, mammographic abnormality, or other breast symptom who were found to have breast cancer | Age and long-term area-of-residence matched controls selected from the National Population Register and interviewed in parallel with the cases | Cases: 98% of those contacted; which is 86% of those potentially eligible. Controls were selected individually for each case (response rate not available) | 23 - 92 |
| Kathleen Cuningham Foundation Consortium for research into Familial Breast Cancer/Australian Ovarian Cancer Study | KConFab/AOCS | Australia and New Zealand | mixed | Cases were from multiple-case breast and breast-ovarian families recruited though family cancer clinics from across Australia and New Zealand from 1998 to the present. Cases were selected for inclusion in BCAC studies if (i) family was negative for mutations in BRCA1 and BRCA2 (ii) case was the index for the family, defined as youngest breast cancer affected family member. | Female controls were ascertained by the Australian Ovarian Cancer Study identified from the electoral rolls from all over Australia from 2002-2006. | 78% family members approached agreed to participate. Of those, 97% provided a blood sample and 96% provided questionnaire data | 19 - 78 |
| Mammary Carcinoma Risk Factor Investigation | MARIE | Germany | population-based | Incident and prevalent cases diagnosed from 2001-2005 in the study region Hamburg in Northern Germany, and from 2002-2005 in the study region Rhein-Neckar-Karlsruhe in Southern Germany. | 2 controls per case were randomly drawn from population registries and frequency matched by birth year and study region to the case. Controls were recruited from 2002 to 2006. | 64.1% of cases & 43.4% of controls participated (rates for people with QX) | 50 - 74 |
| Milan Breast Cancer Study Group | MBCSG | Italy | Mixed | Familial and/or early onset breast cancer patients (aged 22-87) negative for mutations in BRCA genes, ascertained in two large cancer centres in Milan from 1996 to 2008. | Healthy blood donors aged 18-71 years, recruited at two blood centres in Milan from 2004 to 2009 | >99% | 21 - 80 |
| Mayo Clinic Breast Cancer Study | MCBCS | USA | Mixed | Incident cases residing in 6 states (MN, WI, IA, IL, ND, SD) seen at the Mayo Clinic in Rochester, MN from 2002-5 | Women without cancer presenting for general medical examination at the Mayo Clinic. Controls were recruited concurrently with cases and were frequency matched to cases on age, ethnicity and county/state | 68% for cases, 77% for controls were interviewed and provided a blood sample | 22 - 89 |
| Melbourne Collaborative Cohort Study | MCCS | Australia | Prospective cohort | Incident cases diagnosed within the Melbourne Collaborative Cohort Study during the follow-up from baseline (1990-1994) to 2008 of the 24469 participating women | Random sample of the initial cohort | All incident cases and all the controls in the random sample. DNA available from >95% of the participants. | 30 - 82 |
| Northern California Breast Cancer Family Registry | NC-BCFR | USA | Population-based | Cases included those enrolled in the NC-BCFR as part of Phase I and II recruitment. Incident cases aged <65 years diagnosed between 1995 and 2003 were identified through the SEER cancer registry of the Greater San Francisco Bay Area. All cases likely at increased genetic risk were eligible to enroll in the BCFR (dx at age <35 yrs, personal history of ovarian or childhood cancer, bilateral breast cancer with 1st dx at age <50, family history of breast or ovarian cancer in first-degree relatives). Cases not meeting these criteria were randomly sampled (2.5% of whites, 30% of African Americans, 28% of Hispanics, 38% of Asian Americans). | Controls were identified through random digit dialing conducted from 1999-2000 in the same geographic region. Controls were frequency matched to cases on 5-year age group and race/ethnicity, at a ratio of 1 control per 2 cases. | Cases identified by the cancer registry were screened by telephone to assess study eligibility. Of those contacted (alive, with MD permission and valid address), 90% completed the screening interview. Of cases eligible to enroll in the BCFR, 77% completed the epidemiology and family history questionnaires, and 67% provided a blood or mouthwash sample. Of RDD controls, 60% completed the questionnaires, and 56% provided a biospecimen sample. | 22 - 64 |
| Oulu Breast Cancer Study | OBCS | Finland | Mixed | Consecutive incident cases diagnosed at the Oulu University Hospital between 2000 and 2004. | Healthy, consecutive, anonymous, female Finnish Red-Cross blood donors recruited in 2002 from the same geographical region in Northern Finland. | All of the asked controls, and 71% of all cases treated at the Oulu University Hospital, Department of Oncology during the collection period. | 28 - 92 |
| Ontario Familial Breast Cancer Registry | OFBCR | Canada | Population-based | Cases diagnosed between 1 Jan 1996-31 Dec 1998 were identified from the Ontario Cancer Registry which registers >97% of all cases residing in the province at the time of diagnosis. All women with invasive breast cancer aged 20–54 years who met the OFBCR definition for high genetic risk (family history of specific cancers particularly breast and ovarian, early onset disease, Ashkenazi ethnicity or a diagnosis of multiple breast cancer) were asked to participate by completing risk factor questionnaires and providing a blood sample. A 25% random sample of individuals in this age category who did not meet the OFBCR definition, 35% of those aged 55–69 at high risk and 8.75% aged 55–69 at low risk were also asked to participate. This multi-step sampling scheme enriched the population for genetically predisposed individuals, which was an objective of the Ontario Familial Breast Cancer Registry. | Unrelated, unaffected population controls were recruited between 2003-2005 by calling randomly selected residential telephone numbers throughout the same geographical region. Eligible controls were women with no history of breast cancer and were frequency-matched by 5-year age group to the expected age distribution of cases. Approximately, 65% of identified eligible women returned questionnaires, and 63% of these donated a blood specimen. | Cases: consent to contact patients was 92%, response to initial family history questionnaire was 65%, response to risk factor questionnaires was 73% of all eligible, and donation of a blood sample was 63% of all eligible. Less than 2% died before initial contact. Controls: approximately, 65% of identified eligible women returned questionnaires, and 63% of these donated a blood specimen. | 22 - 81 |
| Sheffield Breast Cancer Study | SBCS | UK | Mixed | Women with pathologically confirmed breast cancer recruited from surgical outpatient clinics at the Royal Hallamshire Hospital, Sheffield, 1998 – 2005; cases are a mixture of prevalent and incident disease | Unselected women attending the Sheffield Mammography Screening Service between Sep 2000 - Aug 2004, if their mammograms showed no evidence of a breast lesion | Not recorded | 29 - 93 |
| Study of Epidemiology and Risk factors in Cancer Heredity | SEARCH | UK | Mixed | 2 groups of cases identified through East Anglian Cancer Registry; 1) prevalent cases diagnosed age <55 from 1991-6 and alive when study started in 1996; 2) incident cases diagnosed age < 70 diagnosed after 1996 | Two groups of controls: (1) selected from the EPIC-Norfolk cohort study of 25,000 individuals age 45-74 recruited between 1992 and1994, based in the same geographic region as cases; (2) selected from GP practices from March 2003 to present, frequency matched to cases by age and geographic region | 64% of eligible cases and 41% of invited controls provided a blood sample | 23 - 69 |
| Seoul Breast Cancer Study | SEBCS | Korea | Hospital-based | Consecutive, incident, cases from 2 hospitals in Seoul recruited 2001-2005 | Healthy community controls from same catchment area and participating in annual health check-up, 2001-2005. | ~ 85% of cases, 75% of controls were interviewed and provided a blood sample. | 19 - 89 |
| IARC-Thai Breast Cancer Study | TBCS | Thailand | Hospital-based | Incident cases diagnosed at the National Cancer Institute (NCI) in Bangkok and Khon Kaen Hospital during the period May 2002-March 2004. | Controls were randomly selected healthy females visiting hospital patients with diseases other than breast or ovarian cancer at NCI Bangkok and Khon Kaen Hospital during the period May 2002-March 2004. | 94% of cases and 73% of controls completed a questionnaire and provided a blood sample | 17 - 81 |
| UCI Breast Cancer Study | UCIBCS | USA | Population-based | All cases diagnosed in Orange County, California, during one-year period beginning March 1, 1994. Ascertained through the population-based Cancer Surveillance Program of Orange County California (CSPOC) | Female controls under age 75 years without history of cancer recruited using random digit dialing among Orange County residents & frequency matched to cases by age & race/ethnicity. Recruited from 1998-2003 | Cases 76% and Controls 80% | 24 - 90 |

**Supplementary Table 2.** **Availability of histopathology information for cases (white European women only)**

| **Study Acronym** | **ER** | **PR** | **HER2** |
| --- | --- | --- | --- |
|  |  |  |  |
| ABCFS | 1017 (0.66) | 1014 (0.69) | -- |
| ABCS | 958 (0.66) | 953 (0.52) | 919 (0.25) |
| BBCC | 765 (0.71) | 764 (0.64) | 653 (0.36) |
| BBCS | -- | -- | -- |
| BIGGS | 781 (0.73) | 603 (0.75) | -- |
| CGEMS | 900 (0.79) | 878 (0.69) | -- |
| CNIO-BCS | 242 (0.75) | 260 (0.57) | 126 (0.54) |
| GC-HBOC | -- | -- | -- |
| GENICA | 963 (0.78) | 961 (0.70) | 678 (0.28) |
| GESBC | 424 (0.61) | 417 (0.58) | -- |
| HABCS | 711 (0.89) | 639 (0.88) | -- |
| HMBCS | -- | -- | -- |
| KARBAC | 427 (0.83) | 370 (0.76) | -- |
| kConFab/AOCS | 248 (0.69) | 220 (0.70) | -- |
| KBCP | 434 (0.76) | 432 (0.63) | 394 (0.13) |
| MARIE | 2637 (0.79) | 2635 (0.66) | 1437 (0.20) |
| MBCSG | 288 (0.76) | 286 (0.69) | 177 (0.55) |
| MCBCS | 1723 (0.84) | 1722 (0.75) | 1139 (0.15) |
| MCCS | 553 (0.76) | 547 (0.67) | 224 (0.26) |
| NC-BCFR | 256 (0.84) | 255 (0.73) | -- |
| OBCS | 544 (0.80) | 543 (0.70) | 544 (0.14) |
| OFBCR | 903 (0.71) | 885 (0.61) | -- |
| SBCS | 724 (0.77) | 327 (0.56) | 349 (0.08) |
| SEARCH | 3057 (0.80) | 2081 (0.70) | 1421 (0.11) |
| UCIBCS | 695 (0.80) | 684 (0.70) | -- |
|  |  |  |  |
| **Total** | **19,250 (0.76)** | **17,476 (0.68)** | **8,061 (0.25)** |
|  |  |  |  |

ER, estrogen receptor status known (% positive); PR, progesteron receptor status known (% positive); HER2, human epidermal growth factor receptor 2 status known (% positive)

**Supplementary Table 3.** **Genotypes for rs1607237 by study site**

|  | **Cases** | | | **Controls** | | |  |  |
| --- | --- | --- | --- | --- | --- | --- | --- | --- |
| **Study Acronym** | **TT** | **TC** | **CC** | **TT** | **TC** | **CC** | **MAF** | ***P*-HWE** |
|  | | |  |  |  |  |  |  |
| **Studies of white European women** | | |  |  |  |  |  |  |
|  |  |  |  |  |  |  |  |  |
| ABCFS | 408 | 581 | 208 | 160 | 204 | 69 | 0.395 | 0.768 |
| ABCS | 548 | 674 | 241 | 200 | 268 | 80 | 0.391 | 0.522 |
| BBCC | 391 | 490 | 164 | 347 | 478 | 156 | 0.403 | 0.686 |
| BBCS | 451 | 523 | 178 | 289 | 410 | 128 | 0.403 | 0.379 |
| BIGGS | 353 | 513 | 188 | 268 | 453 | 173 | 0.447 | 0.455 |
| CGEMS | 2020 | 2630 | 786 | 1905 | 2470 | 905 | 0.405 | 0.032 |
| CNIO-BCS | 352 | 317 | 76 | 353 | 364 | 97 | 0.343 | 0.831 |
| GC-HBOC | 312 | 425 | 121 | 440 | 587 | 184 | 0.394 | 0.607 |
| GENICA | 368 | 467 | 152 | 358 | 487 | 150 | 0.395 | 0.456 |
| GESBC | 214 | 256 | 89 | 216 | 267 | 74 | 0.373 | 0.550 |
| HABCS | 378 | 488 | 180 | 329 | 502 | 167 | 0.419 | 0.294 |
| HMBCS | 571 | 822 | 367 | 325 | 487 | 203 | 0.440 | 0.402 |
| KARBAC | 237 | 400 | 168 | 266 | 433 | 159 | 0.438 | 0.459 |
| kConFab/AOCS | 203 | 279 | 84 | 307 | 447 | 140 | 0.407 | 0.280 |
| KBCP | 99 | 237 | 149 | 105 | 215 | 106 | 0.501 | 0.846 |
| MARIE | 1032 | 1298 | 422 | 1985 | 2509 | 808 | 0.389 | 0.743 |
| MBCSG | 329 | 328 | 79 | 516 | 580 | 130 | 0.343 | 0.078 |
| MCBCS | 662 | 825 | 292 | 529 | 745 | 274 | 0.418 | 0.676 |
| MCCS | 250 | 322 | 105 | 270 | 362 | 109 | 0.391 | 0.488 |
| NC-BCFR | 147 | 171 | 67 | 40 | 89 | 25 | 0.451 | 0.038 |
| OBCS | 126 | 292 | 126 | 123 | 259 | 126 | 0.503 | 0.657 |
| OFBCR | 438 | 561 | 167 | 115 | 141 | 73 | 0.436 | 0.020 |
| SBCS | 420 | 606 | 176 | 401 | 579 | 209 | 0.419 | 1.000 |
| SEARCH | 2350 | 3122 | 1020 | 2418 | 3265 | 1056 | 0.399 | 0.400 |
| UCIBCS | 327 | 477 | 145 | 202 | 229 | 90 | 0.393 | 0.074 |
|  |  |  |  |  |  |  |  |  |
| **Studies of Asian women** | |  |  |  |  |  |  |  |
|  |  |  |  |  |  |  |  |  |
| SEBCS | 138 | 644 | 929 | 103 | 446 | 617 | 0.720 | 0.085 |
| TBCS | 42 | 203 | 192 | 34 | 119 | 128 | 0.667 | 0.438 |
|  |  |  |  |  |  |  |  |  |

MAF, minor (C) allele frequency in controls; *P*-HWE, p-value for departure from Hardy Weinberg equilibrium in controls.

**Supplementary Table 4.** Rs1607237 and risk of invasive breast cancer among Caucasians by ER/PR/HER2 status

|  | **N Participants** | | **Log-additive Model** | | **Case-only *P*-heterogeneity** |
| --- | --- | --- | --- | --- | --- |
|  | **Cases** | **Controls** | **OR (95%CI)** | ***P*** |
| ER Status | | | | | |
| ER+ | 14,713 | 28,112 | 0.98 (0.96 – 1.01) | 0.269 |  |
| ER - | 4,371 | 28,112 | 1.01 (0.97 – 1.06) | 0.639 | 0.344 |
| PR Status | | | | | |
| PR+ | 11,794 | 28,112 | 0.99 (0.95 – 1.02) | 0.370 |  |
| PR- | 5,537 | 28,112 | 1.00 (0.95 – 1.04) | 0.812 | 0.942 |
| ER/PR Status | | | | | |
| ER+/PR+ | 10,972 | 28,112 | 0.98 (0.95 – 1.01) | 0.255 |  |
| ER+/PR- | 2,190 | 28,112 | 0.99 (0.93 – 1.06) | 0.797 |  |
| ER-/PR+ | 737 | 28,112 | 1.03 (0.92 – 1.14) | 0.611 |  |
| ER-/PR- | 3,290 | 28,112 | 1.00 (0.95 – 1.05) | 0.991 | 0.842 |
| ER, PR and HER2 Status | | | | | |
| (ER+ or PR+)&HER2- | 5,171 | 21,017 | 0.98 (0.94 – 1.03) | 0.456 |  |
| (ER+ or PR+)&HER2+ | 1,059 | 21,017 | 1.00 (0.91 – 1.10) | 0.995 |  |
| ER-/PR-/HER2- | 994 | 21,017 | 0.97 (0.88 – 1.06) | 0.462 |  |
| ER-/PR-/HER2+ | 535 | 21,017 | 0.96 (0.85 – 1.09) | 0.562 | 0.941 |
| OR, odds ratio; CI, confidence interval; ER, estrogen receptor; PR progesterone receptor; HER2, human epidermal growth factor receptor 2; +, positive; -, negative. | | | | | |
